# Desert Springs: Deep Phylogeographic Structure in an Ancient Endemic Crustacean (*Phreatomerus latipes*)

**DOI:** 10.1371/journal.pone.0037642

**Published:** 2012-07-17

**Authors:** Michelle T. Guzik, Mark A. Adams, Nicholas P. Murphy, Steven J. B. Cooper, Andrew D. Austin

**Affiliations:** 1 Australian Centre for Evolutionary Biology and Biodiversity, School of Earth and Environmental Science, The University of Adelaide, North Terrace, Adelaide, South Australia, Australia; 2 Evolutionary Biology Unit, South Australian Museum, North Terrace, Adelaide, South Australia, Australia; 3 Department of Genetics, School of Molecular Science, La Trobe University, Bundoora Victoria, Australia; Brigham Young University, United States of America

## Abstract

Desert mound springs of the Great Artesian Basin in central Australia maintain an endemic fauna that have historically been considered ubiquitous throughout all of the springs. Recent studies, however, have shown that several endemic invertebrate species are genetically highly structured and contain previously unrecognised species, suggesting that individuals may be geographically ‘stranded in desert islands’. Here we further tested the generality of this hypothesis by conducting genetic analyses of the obligate aquatic phreatoicid isopod *Phreatomerus latipes.* Phylogenetic and phylogeographic relationships amongst *P. latipes* individuals were examined using a multilocus approach comprising allozymes and mtDNA sequence data. From the Lake Eyre region in South Australia we collected data for 476 individuals from 69 springs for the mtDNA gene *COI*; in addition, allozyme electrophoresis was conducted on 331 individuals from 19 sites for 25 putative loci. Phylogenetic and population genetic analyses showed three major clades in both allozyme and mtDNA data, with a further nine mtDNA sub-clades, largely supported by the allozymes. Generally, each of these sub-clades was concordant with a traditional geographic grouping known as spring complexes. We observed a coalescent time between ∼2–15 million years ago for haplotypes within each of the nine mtDNA sub-clades, whilst an older total time to coalescence (>15 mya) was observed for the three major clades. Overall we observed that multiple layers of phylogeographic history are exemplified by *Phreatomerus*, suggesting that major climate events and their impact on the landscape have shaped the observed high levels of diversity and endemism. Our results show that this genus reflects a diverse fauna that existed during the early Miocene and appears to have been regionally restricted. Subsequent aridification events have led to substantial contraction of the original habitat, possibly over repeated Pleistocene ice age cycles, with *P. latipes* populations becoming restricted in the distribution to desert springs.

## Introduction

Australia’s arid zone, as defined by Byrne *et al*. 2008 [1], comprises one of the largest desert regions in the world. Despite its low average rainfall of 100–250 mm per year, this region nevertheless harbours a multitude of diverse and endemic faunal groups, such as lizards [2], birds [3], ants [4], and even aquatic animals [5], [6]. The origins of this biome date back to the late Tertiary, since central Australia was considered “warm and wet” until the Miocene [1], [7]. However, two periods of aridification during the Late Miocene and subsequently the early Pliocene and Pleistocene [7], [8], [9] are thought to have led to significant contraction of mesic habitats, with major evolutionary consequences for its inhabitants. In aquatic habitats especially, fragmentation and the resultant isolation of populations is believed to have occurred during aridification, leading to a suite of relictual fauna and high levels of endemism (e. g. [1], [10].

Phylogeographic studies have played a major role in helping untangle the origins of this diversity and the impact of aridification processes on the evolution of Australian taxa (reviewed in [1], [11]). In particular, several studies have revealed high levels of unacknowledged species richness and shared biogeographic histories of fauna within remnant aquatic habitats [12], [13], [14], [15]. However, the immensity and complexity of Australia’s arid region means that additional phylogeographic studies are required for other taxa that have survived aridification before we can fully understand the impact of aridity and the nature of the environment that preceded it. Reflecting this need, extant taxa found in relictual, aquatic, arid-land ecosystems are ideally suited for this purpose, since they have survived despite enduring arguably the most dramatic climate shifts experienced by any of the desert faunas [16].

**Figure 1:**
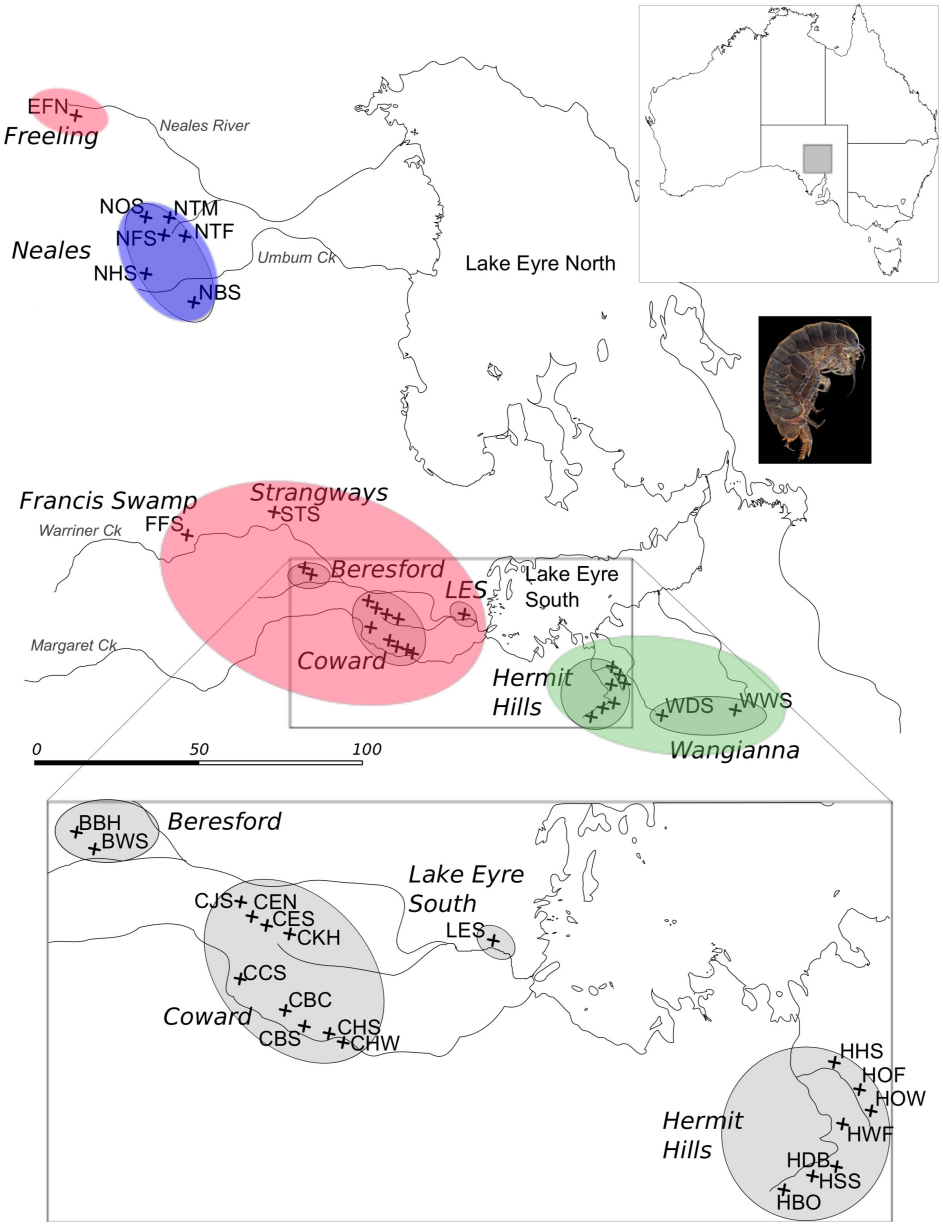
Map of Australian Southern Lake Eyre GAB springs by river catchment (named), spring complex (named) and spring group (denoted by crosses and abbreviated names). Colours denote three major clades observed in mtDNA and allozyme data (Figures 2, 3 and 4) that match geographic regions: Red = ‘Central’, Blue = ‘Northern’, Green = ‘Southern’. Inset is the location of springs relative to Australian continent and image of study organism *Phreatomerus latipes*.

A remnant aquatic habitat that continues to harbour thriving populations of relict fauna from a wetter period in Australia’s history is a distinctive spring super-system known as Great Artesian Basin (GAB) mound springs in the stony desert of South Australia. These groundwater-fed, island-like, wetland habitats are surrounded by sparse desert and sit on the edges of Australia’s largest inland catchment, the Lake Eyre Basin (Fig. 1). Their characteristic mound shape is formed when water is released from the GAB to the surface via geological pressure points where minerals and carbonates are deposited. Protected federally as an ‘endangered ecological community’ (Commonwealth Environmental Protection and Biodiversity Conservation Act 1999), these habitats harbour a suite of endemic spring species including plants [17], vertebrates (e.g. fish [18]) and invertebrates (e.g. snails [19] and especially crustaceans [20], [21]). Springs on the southern side of Lake Eyre (i.e. Lake Eyre supergroup) are some of the most intact and least disturbed environmentally. These Lake Eyre springs are the geographic focus of the current study.

Early studies of endemic aquatic GAB spring taxa suggested that several endemic monotypic genera existed throughout the entire range of these mound springs [22]. However, recent morphological and genetic appraisals have shown that several of the GAB spring endemic taxa comprise numerous phylogenetic lineages, each generally confined to geographically isolated groups of springs (i.e., snails [19], [23], [24], amphipods [15], [25], [26] and wolf spiders [27]). Nevertheless, temporal frameworks for this ecosystem remain rare, with molecular clock estimates of the divergence time among incipient species being investigated for amphipods [15] and more recently snails [28]. In those studies, lineage diversification was estimated to coincide with post-Miocene aridification of the Australian continent [15]. Under this scenario, previously widespread species in rainforest environments of the Miocene are thought to have become stranded in isolated groundwater-fed wetlands, following the drying of inland swamps and lakes. Thereafter, these relict populations persisted throughout the Plio-Pleistocene and, as a direct consequence of the isolation and contraction of their habitats and adaptation to their modified spring environments, experienced *in situ* genetic diversification followed by allopatric speciation. Under this view, groups of GAB springs are equivalent to aquatic islands in a surrounding “sea” of desert. A similar hypothesis has been proposed to explain the diverse subterranean invertebrate fauna (e.g. amphipods [12]; bathynellids [29]; beetles [30], [31], [32]; isopods [14]) in groundwater calcretes of arid Western Australia. Our primary question therefore, is whether there is a possibility that widespread species, in the strict sense, exist throughout the mound spring region.

Relicts, as defined by Habel *et al*. [33] describe descendents of a once widespread fauna that currently have a narrow geographic distribution, and often originate from wide-scale climate and environment changes. One possible relict taxon of post-aridification isolation and subsequent diversification in the Lake Eyre GAB springs is the endemic phreatoicid isopod, *Phreatomerus latipes* Nicholls 1924. A monotypic genus, *P. latipes* is one of the largest (1–2 cm) and most conspicuous endemics restricted solely to these springs. With no congeners, *P. latipes* is considered a biogeographic relict species in the true sense of the term. Interestingly, *P. latipes* is considered to be ‘widespread’ throughout the southern Lake Eyre mound springs [34], inferring that gene flow exists among all populations throughout its range. However, as an obligate aquatic invertebrate without a dispersive life stage [35], *P. latipes* does not appear capable of dispersal across the desert landscape in the absence of aquatic connections [36], [37]. As such, *P. latipes* may exhibit strong genetic sub-structuring in this region [15], [38]. Alternatively, dispersal of *P. latipes* among GAB springs may occur along ephemeral river systems. The springs are located on the edges of the large ephemeral river drainage catchment, the Lake Eyre Basin. Water feeds into this giant inland lake from tributaries coming from all directions and it has been a permanent water feature at various times in Australia’s history [39], [40]. Today, the saline Lake Eyre is only close to full during major monsoonal events in Australia’s north, at which time the inland rivers flow. The impact of these seasonal river systems on mound springs fauna are yet to be investigated in detail, but broader scale studies of inter-specific relationships have found that closely related species are often clustered geographically in line with river catchments [15], [19], [23], [26], [27]. Five primary river systems are known to intersect the mound springs prior to arriving in Lake Eyre: Margaret Creek, Warriner Creek, Neales Creek, Hermit Hills drainage (numerous tributaries) and Umbum Creek. These river drainages may provide vital aquatic dispersal corridors between otherwise fragmented mound springs habitats. Therefore, any studies of phylogeographic structure amongst mound spring populations must consider river drainages (e.g. [41]).

Here we used a multilocus genetic approach using mtDNA and allozymes to investigate the phylogenetic and phylogeographic history of *P. latipes* throughout the highly fragmented Lake Eyre mound springs. Our aim was to investigate whether major climatic events during the history of the Australian arid region have impacted on the spatio-temporal history of a single, supposedly widespread species from freshwater springs in the Australian desert. Based on previous studies of spring invertebrates we hypothesised that a) major periods of aridification are likely to have isolated taxa in the GAB mound springs, in particular during the period of aridification following the late Miocene after which there was a return to wet and the subsequent Pliocene aridification and b) the island-like GAB mound springs have promoted a high degree of genetic isolation among spring groups, leading to substantial genetic diversification within *P. latipes*.

## Materials and Methods

### Ethics Statement

None of the aforementioned field studies involved endangered or protected species, all sites were on private land or in National Parks, and we comprehensively obtained permission to access and sample all sites. For collections in National Parks we obtained a permit to ‘undertake scientific research’ (permit number Z25519 to Dr Nicholas Murphy) using appropriate methods and ethics approval from The Government of South Australia, Department for Environment and Heritage signed for the Minister for Environment and Conservation. For collection on private property we obtained general permission from Greg Campbell (Chief Executive Officer) of S. Kidman & Co Ltd and also directly from a number of station managers to carry out our field collections. The station managers were: Randall Crozier for Anna Creek Station, Peter Paisley for Stuart Creek Station, Bobby Hunter for The Peak Station. We also received permission to access culturally sensitive land at Hermit Hills Springs (Reg Dodd). At the privately owned properties we used the same methods as those in the national parks.

### Animals and the Environment

Little biological information (i.e. desiccation tolerance, habitat preference, etc.) is available for *P. latipes*. The species is known to brood ∼10 live young, born as miniature adults, in a pouch carried by the female [35]. The taxon is also restricted to freshwater for respiration. Population sizes within flowing springs are typically very high throughout the spring habitat, although little is known about the effective population sizes and their susceptibility to environmental change. Springs vary in size from large wetlands (300 m^2^) to small seeps (0.5 m^2^) both of which may contain endemic invertebrates. This study was undertaken on the Lake Eyre supergroup of GAB mound springs, located throughout the southern and western portion of the Lake Eyre Basin in central Australia (Fig. 1). These individual springs occur around areas of geological weakness (i.e. faults) and as such form large clusters of directly connected spring outlets, known as *spring groups* (Fig. 1). These spring groups can be hierarchically categorized further as *spring complexes* (Fig. 1) based on their hydrogeological and broader geographic location.

Despite relying on groundwater flows for spring existence, the ephemeral rivers and streams of the Lake Eyre Basin may provide an aquatic medium for connectivity between fragmented spring habitats. Major river drainages flow from a number of directions into Lake Eyre, a giant (∼9,690 km^2^) inland lake that infrequently receives water during major rainfall events, and historically contained large amounts of water [39], [40]. The extant river drainages are shown in Fig. 1 and can be used to delineate the spring complexes. The major drainages and the spring complexes that they potentially connect are: Margaret Creek (Billa Kalina, Coward spring complexes (excluding Elizabeth North, South, Jersey, Kewson Hill spring groups), Lake Eyre South); Warriner Creek (Elizabeth North, South, Jersey, Kewson Hill spring groups, Beresford, Francis Swamp Lake Eyre South, Strangways spring complexes); Umbum Creek (Neales River spring complex); Neales Creek (Freeling spring complex). Finally, the spring complexes of Hermit Hills and Wangianna are independently connected to several tributaries that intersect at Lake Eyre.

Individuals were collected using fine mesh nets during 2008–9. Nets were rinsed with water and ethanol between springs to prevent contamination and all specimens were stored in 100% ethanol. Not all springs yielded *P. latipes,* but most did in high numbers. Some samples used in this study were obtained from ‘historical’ archived material from the frozen tissue collection of the South Australian Museum. These specimens were collected in 1985 and kept at −80°C since that time. Allozyme analyses were conducted solely on these frozen samples and were completed by 1987. However, the limited number of historic sites plus a lack of precision in their geographic coordinates necessitated the collection of fresh material for mtDNA analysis. Any discrepancies observed between the allozyme and mtDNA profiles of a spring group were explored by further sequencing a selection of the ‘historical’ samples. Finally, an ‘historical’ representative from most key clades was sequenced to ensure consistency of results between the disparate (>20 years) sampling periods.

### DNA Isolation and Amplification of Mitochondrial DNA

We used partial DNA sequences of the mtDNA gene, *Cytochrome Oxidase subunit 1* (*COI*) to examine the phylogeographic structure of *P. latipes*. This mtDNA gene has been used to successfully elucidate phylogeographic and population genetic relationships within crustacean taxa in previous studies (e.g. [42]). DNA was extracted from legs or whole animals using Chelex beads according to the standard protocol [43]. A 597 base pair (bp) region of the COI gene was amplified with the new primers M1070 (Forward) (5′TATTTTGTAYTAGGATCATGAGCGGGTG3′) and M1058 (Reverse) (5′CCTAAAATWCCAATTCCRATTATTGC3′) and crustacean primers LCO1490 (5′-GGTCAACAAATCATAAAGATATTGG-3′) and HCO2198 (5′-TAAACTTCAGGGTGACCAAAAAATCA-3′) [44]. Polymerase Chain Reaction (PCR) amplification of all sequences involved an initial cycle of denaturation at 95°C for 2 min, and 35 subsequent cycles of 94°C for 30 seconds (s), 50°C for 30 s and 72°C for 1 min. PCR was carried out in 25 µl reactions containing 10×Eppendorf Hotmaster® Taq Buffer (Eppendorf, Westbury, NY, USA) containing 2.5 mM Mg^2+^, 2.5 mM of each dNTP, 5.0 µM of each primer, 0.1 units of Eppendorf Hotmaster® Taq Polymerase and ∼1 ng of DNA. These PCR products were sequenced using the ABI PRISM Big Dye Terminator Cycle Sequencing kit (Applied Biosystems, Foster City, CA, USA) and the ABI PRISM 3700 DNA analyzer. All sequences were edited with reference to chromatograms using BioEdit version 7.0.1 [45] and aligned using Clustal W [46]. A number of Genbank sequences were also added to the data set from Hermit Hills and Wangianna spring complexes: PLH1-29 amd PLD1, 2 (HM068160-91) [26].

Outgroup sequences for phylogenetic analysis were chosen from the same family as *Phreatomerus* (Phreatoicidea: Amphisopidae), namely *Amphisopus lintoni* Nicholls 1924 (Genbank accession EF203063 [47]) and one haplotype of *Paramphisopus palustris* Glauert, 1924 (Genbank accession EF203022 [47]). However, the higher level relationships of genera within the family Amphisopididae, whilst recently revised [48], are yet to be formally explored using DNA sequence data and the long branches of these outgroups confounded our own assessments of fine-scale phylogeography within *Phreatomerus*. Therefore, subsequent analyses employed unrooted trees, although a rooted tree is presented for completeness in Figure S1.

### Phylogenetic Analysis of mtDNA Sequences

The phylogenetic relationships among individual *COI* haplotypes were analysed using a Bayesian approach, as implemented with MrBayes 3.1.2 [49]. The model that best fitted the data was estimated with Modeltest 3.7 [50] for nucleotide data under an Akaike Information Criterion framework. Models were tested for all three codon positions; the GTR+G model was favoured for first, F81 for the second, and GTR+G for the third position. The nucleotide sequence data were partitioned by codon position and each partition was started independently with a different model (listed above). All parameters were unlinked and the rates were allowed to vary over the partitions. Four chains were run simultaneously for 10,000,000 generations in two independent runs, sampling trees every 100 generations. To evaluate convergence to the stationary distribution the program Tracer 1.4 [51] was used. The likelihood values converged to relative stationarity after about 10,000 generations. A burn-in of 10,000 was chosen and a 50% consensus tree was constructed from the remaining trees.

ARLEQUIN v.3.1 [52] was used to carry out population analyses of diversity and the demographic history, by computing Fu’s F_s_ (*F_s_*) [53], Tajima’s D (*D*) [54], parameters for the model of population expansion (time since expansion (τ), and relative population sizes before (θ_0_) and after (θ_1_) expansion) and for the continent-island model of demographic expansion (τ = 2 *Tμ*, θ = 2 *Nμ* and *M = *2 *Nm*, where *T* = number of generations before spatial expansion, *μ* = mutation rate, *N* = size of deme (assumed constant) and *m* = fraction of individuals from a deme exchanging with other demes). The generalized least-squares approach [55] in ARLEQUIN was used to test the empirical mismatch distribution against a model of Demographic or Spatial expansion.

To examine the relationships among haplotypes more closely than the phylogenetic approach used above, we estimated haplotype networks within each of the nine sub-clades. MtDNA *COI* sequences were analysed using a parsimony approach with TCS v.1.21 [56] to generate and arrange haplotype networks at a 95% connection limit.

### Estimating Coalescent Time of mtDNA Sequences

BEAST v1.4.7 [57] was used to estimate the coalescence time of *COI* sequences among sub-clades and major clades. The subprogram BEAUti v1.4.7 [57] was used to create input.xml files, and Tracer v1.4 [51] was used to analyse the parameter distributions estimated from BEAST. An UPGMA starting tree was estimated under the HKY+I+G model in which (a) base frequencies were estimated, (b) codon positions were partitioned (positions 1+2, 3) and (c) the parameters, substitution model across codon positions and rate heterogeneity model were unlinked. The substitution rate was fixed at 0.0115 (standard arthropod mtDNA molecular clock of 2.3% divergence per million years [58]), and a relaxed clock (uncorrelated lognormal) was used. A number of different tree prior models were subsequently implemented separately on the complete *Phreatomerus* data set using BEAST (for example, constant, exponential, logistic, expansion and Yule speciation). Each analysis was run for 10,000,000 generations, with sampling every 100 generations, and the burn-in was 25% of the total sampled trees (that is, 25,000). Each analysis was run multiple times and all estimated dates were found to be consistent among different runs.

### Allozyme Laboratory Procedures

Allozyme electrophoresis of whole animal homogenates was undertaken on cellulose acetate gels (Cellogel™) according to the principles and procedures of Richardson *et al*. [59]. The following enzymes or non-enzymatic proteins produced zymograms of sufficient quality to permit allozymic interpretation: ACYC, ARGK, DIA, ENOL, FDP, GOT, GP, GPI, GPT, HK, IPP, MDH, MPI, PEP-A, PEP-B, PEP-C, PEP-D, PGM, PK, SORDH, and TPI. Details of enzyme/locus abbreviations, enzyme commission numbers, electrophoretic conditions, and stain recipes are presented elsewhere [59]. Allozymes were labelled alphabetically and multiple loci, where present, were labelled numerically in order of increasing electrophoretic mobility (e.g. *Acyc* ^a^ < *Acyc* ^b^; *Got1*< *Got2*).

### Allozyme Data Analyses

We initially used Principal CO-ordinates Analysis (PCO) to assess the genetic affinities among all individuals, independent of any *a priori* grouping by spring, spring group, or mtDNA profile. Where discrete PCO groups were evident and diagnosable from one another by fixed differences (i.e. no alleles in common) and/or near-fixed differences (the cumulative frequency of shared alleles is no more than 10%) at two or more loci, individuals within each distinct PCO group were subsequently subjected to a further round of PCO to assess whether significant additional heterogeneity was present in deeper dimensions. Horner & Adams [60] present the rationale and methodological details underlying the use of “stepwise” PCO.

We also undertook a range of standard population genetic analyses, using the procedures and software employed by Horner & Adams [60]. The genotypic data were examined for statistical evidence of any deviation from Hardy Weinberg expectations or linkage disequilibrium within sites, plus any heterogeneity of allele frequencies between sites within each taxon (using the program ‘GENEPOP v3.4’ [61]). All probability values were adjusted for multiple tests using the sequential Bonferroni correction factor. In addition, the genetic affinities among sites were assessed by constructing an unrooted NJ network from a matrix of pairwise Nei D values among sites.

A final set of Bayesian analyses involved using the R statistical package ‘Geneland’ (R, Development Core team, 2005; [62]) to assess the number of discrete subpopulations (k) in those PCO groups represented by more than a single site. A series of 10 replicate runs, one for each of the “uncorrelated” and “correlated” frequency models, was undertaken without imposing limits on k (i.e. k was allowed to vary between 1 and the maximum number of individual springs). Each individual run permitted the existence of null alleles and involved 100,000 iterations, with thinning every 1000 iterations. As discussed by Guillot [62], the “correlated frequency” model is likely to be more sensitive in detecting subtle population differentiation, but may also be more prone to algorithm instabilities. As there was no evidence of the latter in our analyses, we ultimately chose this model for determining the value of k.

## Results

### Phylogenetic Analyses Based on MtDNA

Overall 597 bp of the mtDNA gene *COI* were sequenced for 476 individuals from a total of 69 springs from southern Lake Eyre, South Australia (Table S1). Ninety-two unique haplotypes were detected (labelled with the initial of the spring complex): **B**eresford (B1–B2) **C**oward (C1–C2, C6–C25,); one haplotype shared between **B**eresford and **C**oward (BC1); **L**ake Eyre South (L1–L2); Billa **K**alina (K1); **N**eales (N1–N10, N12–N15, N17–N21, N23); **H**ermit Hills (H1–H20, H22–H27, H29, H31); **D**avenport (D1–D6); **FRA**ncis Swamp only haplotypes (FRA1–FRA3); **S**trangways (S1); one haplotype shared between **F**rancis Swamp + **S**trangways (FS1); and **FRE**eling (FRE1–FRE5). All haplotype sequences have been submitted to Genbank (JQ612592–JQ612655).

**Figure 2:**
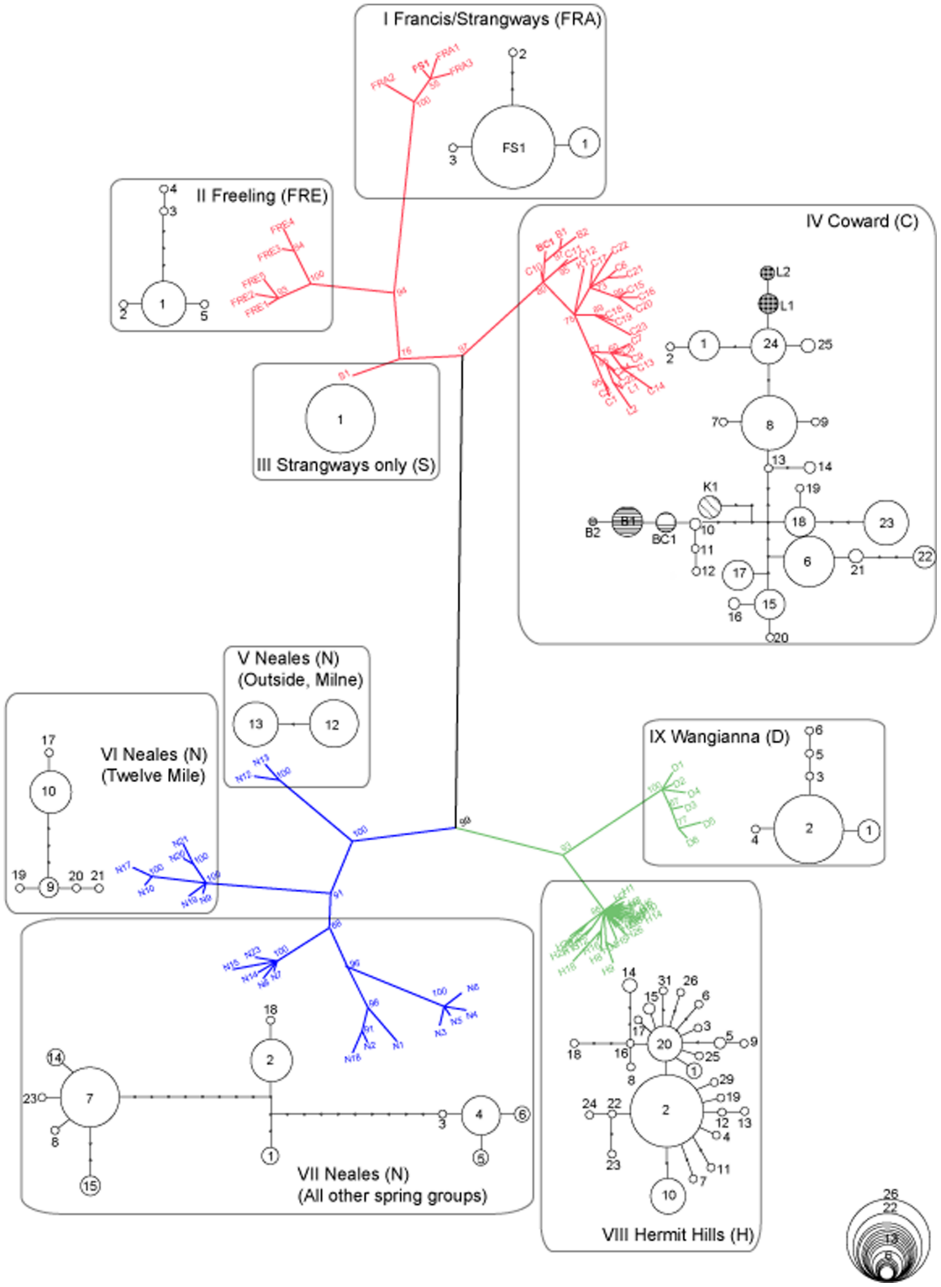
Consensus phylogeny estimated using a Bayesian approach that employed the GTR +I +G model of nucleotide evolution for haplotypes of *Phreatomerus latipes*. Posterior probability support is listed as a percentage next to the corresponding node. Lists of specimens and haplotypes are listed in Table S1. Colours denote three major clades that match geographic regions shown in Figure 1: Red = ‘*C*entral’, Green = ‘*S*outhern’, Blue = ‘*N*orthern’ and subclades I-IX (boxed) represent phylogenetic structure amongst haplotypes. Bold font represents haplotypes shared between spring complexes (FS1 and BC1). Corresponding haplotype networks for each of the sub-clades are shown and haplotypes with a hatched (Lake Eyre South complex), straight line (Beresford complex) or horizontal line (Billa Kalina complex) patterns differentiate distinct spring complexes from the Coward complex.

The phylogenetic analysis of *COI* haplotypes among *P. latipes* of the mound springs revealed three primary clades, hereafter referred to by their rough geographic position relative to Lake Eyre: C (Central), S (Southern) and N (Northern) (Fig. 2). While clades S and N were geographically restricted to single regions, clade C was present in all central springs plus the geographically disjunct Freeling spring complex, the most northerly of all the spring complexes. The mtDNA tree further partitioned these primary clades into nine major sub-clades (I-IX, Fig. 2), most of which were either concordant with the traditional geographic grouping of spring complexes or had connections with adjacent spring complexes or matched spring groups within complexes. Thus clade C comprised four sub-clades (I = Strangways + Francis, II = Freeling, III = unique to Strangways, IV = Coward + Billa Kalina + Beresford + Lake Eyre South, clade N contained three sub-clades (V = Outside + Milne spring groups, VI = Twelve Mile spring group, VII = all other clade N spring groups), and clade S contained two sub-clades VIII (Wangianna) and IX (Hermit Hills).

### Molecular Diversity

Haplotype diversity (H_d_) was generally moderate to high for all spring complexes of *P. latipes* (Table 1) with the lowest estimates observed at Freeling and Beresford (H_d_ = 0.35–0.39). In contrast, nucleotide diversity (polymorphism) represented by the mean number of pairwise differences (π) was consistently low to moderate with the highest nucleotide diversity at Strangways and Neales River (π = 11.5–12.9) even though only two haplotypes were sampled at Strangways.

**Table 1:** Molecular diversity indices and population demographic parameters under two models of population expansion for *Phreatomerus latipes* from spring complexes throughout the Lake Eyre region.

								Demographic expansion				Spatial expansion			
Complex (Sub-clade)	*n*	*h*	*S*	H_d_	π	*D*	*F_s_*	τ	θ_0_	θ_1_	SSD	τ	θ	*M*	SSD
Strangways (I)	38	2	23	0.5±0.03	11.5	3.7	24.6	0	0	Inf.	**0.50**	24.1	0	1.1	**0.22**
Francis Swamp (I)	23	4	5	0.6±0.06	0.9	−1.1	−0.3	0.8	0	Inf.	0.03	0.8	0	Inf.	**0.03**
Freeling (II)	19	5	7	0.4±0.14	1.1	−**1.5**	−0.9	3.0	0	0.6	0.03	4.9	0.4	0.3	0.01
Coward (IV)	126	20	30	0.9±0.01	6.1	0.3	−0.1	8.1	0.005	24.6	**0.02**	7.7	0.	10.0	0.02
Beresford (IV)	11	3	2	0.4±0.17	0.4	−**1.4**	−**1.3**	0.4	0	Inf.	0.00	0.4	0	Inf.	0.00
Lake Eyre South (IV)	8	2	2	0.5±0.12	1.1	1.5	2.1	2.6	0.004	2.2	0.20	2.3	0	2.0	**0.16**
Billa Kalina (IV)	6	1	-	-	-	-	-	-	-	-	-	-	-	-	-
Neales (V+VI+VII)	131	20	45	0.9±0.01	12.9	1.7	6.3	16.7	0.002	50.6	**0.01**	14.7	1.8	7.1	0.01
Wangianna (VIII)	32	5	5	0.5±0.09	0.7	−1.2	−1.6	0.7	0	Inf.	0.01	0.7	0	Inf.	0.01
Hermit Hills (IX)	82	24	31	0.9±0.03	2.4	−**1.9**	−**15.2**	2.8	0	12.3	0.00	2.6	0	14.1	0.00

Abbreviations: *n*, sample size; *h*, number of haplotypes, *S*, number of polymorphic sites; H_d_, gene diversity; π, nucleotide diversity as mean number of pairwise differences in the population; Tajima’s *D*; Fu’s *F_s_*. Model of demographic expansion parameters, where τ is an index of time since the expansion expressed in units of mutational time; θ_0_ and θ_1_ are pre- and post-expansion values for the mutation parameter (that is, 2 Nμ, where N is the effective female population size and μ is the mutation rate per gene per generation); SSD, Sum of Squared Deviations between the observed and the expected mismatch as a test statistic; Model of spatial expansion parameters where: θ, the effective size of each deme; *M*, relative rate of gene exchange between demes; -, not estimated; bold font represents significance tests where *P*<0.05; inf, infinite estimate.

### Haplotype Networks

All nine sub-clades showed typical patterns of expansion (or selection) in haplotype networks where a single dominant haplotype had a few single point mutations leading to novel and rare haplotypes (Fig. 2). Additionally, subclades from clade N were significantly structured but did join the networks at 95% confidence in parsimony analyses (Fig. 2). Importantly, all but two of the 92 haplotypes were restricted to a single spring complex, the exceptions being haplotype FS1 (sub-clade I; shared between Strangways and Francis Swamp across a ∼25 km gap) and haplotype BC1 (sub-clade IV; shared at low frequency over ∼19 km between the Beresford and Coward complexes). However, the majority of haplotypes present in each of these four spring complexes were unique to that complex.

### Historical Demography

Some evidence for a departure from neutrality and population expansion in *COI* was observed in the Lake Eyre GAB mound spring system. Negative and significant estimates of *F_s_*, *D* were observed in Freeling, Beresford and Hermit Hills spring complexes (Table 1). Selection is typically indicated by an excess of identical haplotypes, which is seen in only a few of these populations, although these tests are not able to distinguish between selection/genetic hitch hiking and demographic processes [63]. Estimates of the time since expansion parameter (τ) and relative population sizes before (θ_0_) and after (θ_1_) expansion under the Demographic Expansion model (Table 1) indicated most of the spring complexes showed relatively recent (low) demographic expansion events (τ = 0–8.13), excluding Neales (τ = 16.72) which showed evidence of an older demographic expansion. The Spatial Expansion model showed a similar pattern to the Demographic Expansion model with only Strangways revealing an older spatial expansion overall (τ = 24.12). Each of the spring complexes generally showed spatial expansion in this species with low effective population size and low gene exchange between demes (θ = 0–1.82; *M* = 14.0) with a few exceptions (e.g. Francis Swamp, Beresford, etc.).

### Coalescent Timing

The BEAST analyses all reached convergence, with effective sample size values well above 100. Here we present the results of the Yule model tree prior analyses in Table 2, including the lower and upper bounds of the highest posterior density (HPD) interval (HPD being a credible set that contains 95% of the sampled values). The Yule model tree prior was favoured because it is most suitable for trees describing the relationships between individuals from different species, however results for other tree model priors are shown in Table S2. We observed a total coalescent time of ∼2.2 (0.2–5.1 95% HPD)–12.3 (7.2–17.7 95% HPD) for haplotypes within the nine sub-clades (Table 2 and Table S2), whilst an older total time to coalescence of >15 mya was observed for the three major clades (C, S and N).

**Table 2:** Estimates of time since most recent common ancestor of haplotypes from individual sub-clade/clades of *Phreatomerus latipes* from the Lake Eyre region based on a Yule Prior coalescent model using a Bayesian coalescent approach with BEAST [57].

Clade/Sub-clade	Yule Prior		
	Mean (my)	95% HPD lower	upper
I Francis/Strangways	3.2	0.9	6.0
II Freeling	5.2	1.9	9.1
IV Coward	8.8	5.7	12.6
Clade C (I+II+III+IV) Total	18.7	12.2	25.8
V [ = V Neales1]	2.2	0.2	5.1
VI [ = V Neales2]	12.3	7.2	17.7
VII [ = V Neales3]	4.5	2.1	7.3
Clade N (V+VI+VII) Total	15.7	10.2	21.5
VIII Wangianna	3.8	1.2	6.7
IX Hermit Hills	6.9	4.1	10.0
Clade S (VIII + IX) Total	13.5	8.0	19.6

Both mean date estimates and highest posterior density (HPD) intervals (lower and upper) are presented as numbers of million years (my).

### Allozyme Support for mtDNA Clades

**Figure 3:**
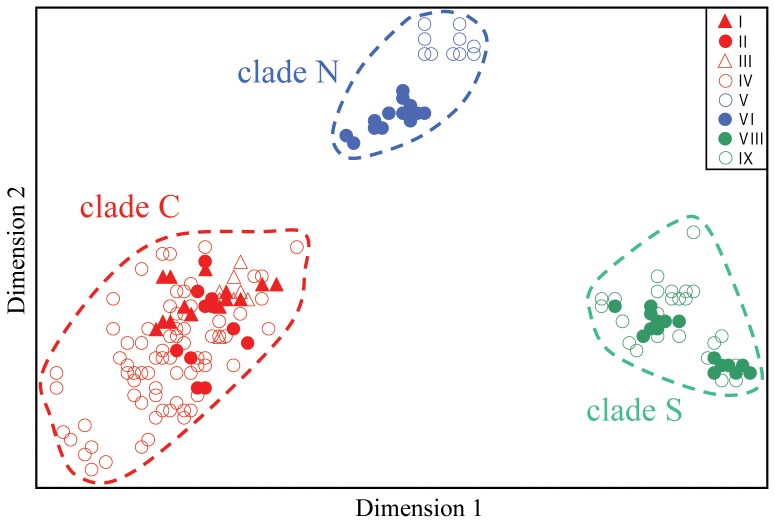
Results of the initial PCO on all 331 individuals. The relative PCO scores have been plotted for the first (X-axis) and second (Y-axis) dimensions, which individually explained 44% and 16% respectively of the total multivariate variation. Individuals are identified using symbols reflecting their mtDNA sub-clade (legend within figure).

**Figure 4:**
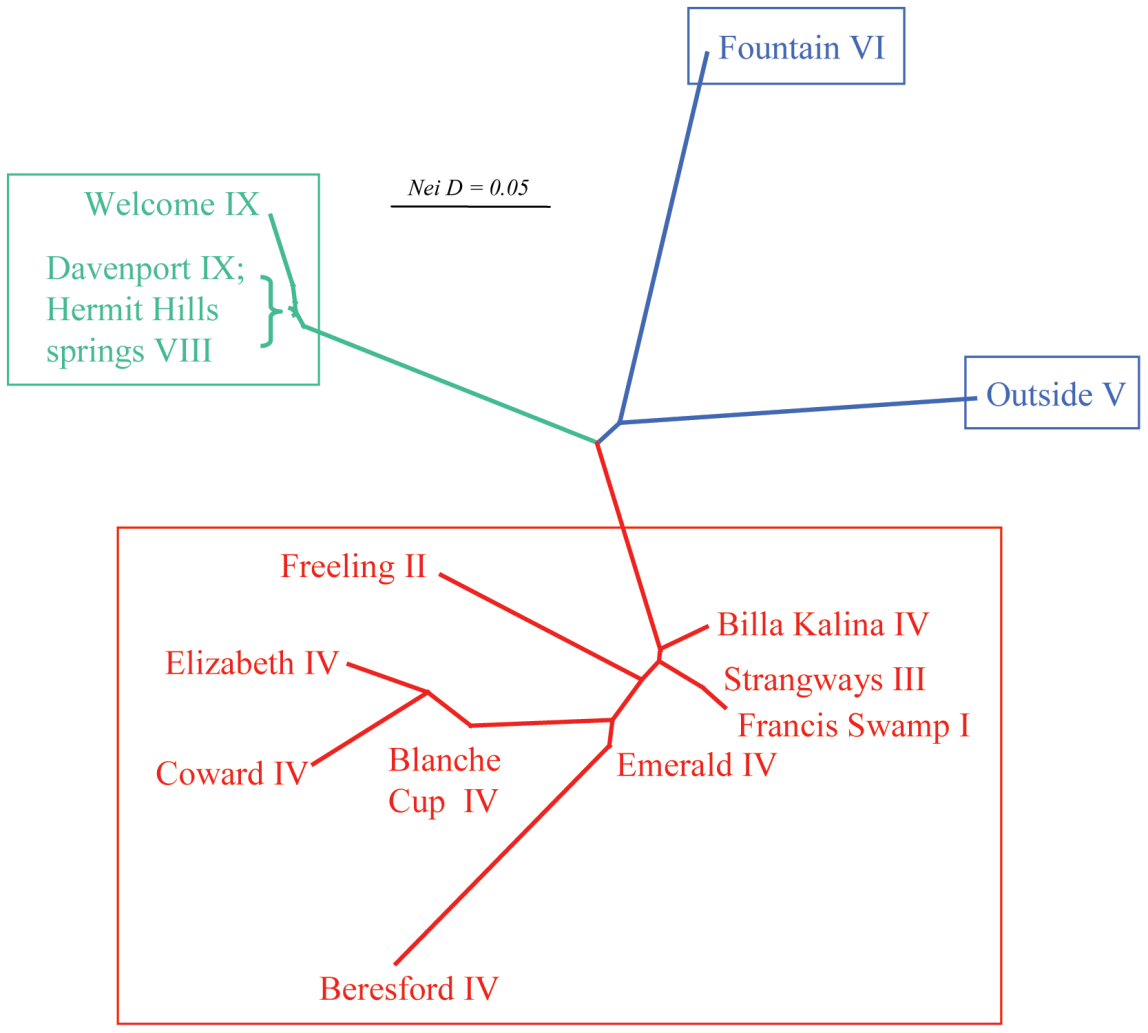
NJ network among sites, based on pairwise Nei Ds. Sites are labelled by spring group and by mtDNA sub-clade, plus coloured by mtDNA clade (as per Fig. 2), as are the branches leading to nodes. The four major taxa identified by PCO as being diagnosable at four or more allozyme loci are also delineated by rectangles.

The final allozyme dataset comprised genotypes at 25 putative loci for 331 individuals from 19 sites. An initial PCO on all individuals (Fig. 3) identified three primary genetic groups, diagnosable from each other by fixed or near-fixed differences at four loci (Table 3) and corresponding to the C, S and N primary mtDNA clades. Henceforth, we refer to the three genetic groups as ‘Clades’, here representing groups of individuals, sharing homologous genetic characteristics and a single common ancestor. Further exploration using stepwise PCO on the individuals within each PCO group provided different outcomes for each primary clade (raw analyses not presented). Clade N comprised two genetically divergent populations, displaying five fixed differences (Table 3) (Outside = V; Fountain = VII; also displayed in Fig. 2). Although some substructure was evident in clade C, its three PCO subgroups differed by only 1–2 near-fixed differences and none were concordant with any of the four mtDNA sub-clades (I-IV). Finally, clade S displayed no obvious PCO sub-groups and no association between mtDNA sub-clade (VIII or IX) and PCO score. In addition to further confirming the extent to which the allozyme and mtDNA profiles of each spring population were concordant, a NJ network among individual springs (Fig. 4) visually demonstrates the disparity in levels of within-clade genetic divergence between clades C, S and N.

**Table 3:** Allele frequencies at all variable loci for the four taxa diagnosed by stepwise PCO.

Locus	Southern (142)	Central (144)	Northern:Fountain (20)	Northern:Outside (25)
*Acyc*	b^99^,a	b	b	b
Dia*	b^97^,a	b	c	d
*Got1*	a	a	a	a
*Got2*	c^98^,e	c^48^,b^46^,d^5^,a	c^62^,b	c^98^,b
Gpi*	c	e^84^,d^10^,c	b	c^92^,a
*Gpt*	b	b^95^,c	b^97^,a	b
Hk*	b	b^99^,a	b	a
*Mdh1*	b	b^66^,a	b	b
Mdh2*	c	b (a <1%)	c	c
Mpi*	d^98^,c^1^,e	c^78^,b^12^,d^9^,a	c	c
*Pep-A*	b^97^,a	b^71^,c^16^,a^12^,d	b	b
*Pep-B*	b^87^,a	b^99^,a	b	b
Pep-C*	a	b^88^,d^6^,c^4^,a	d	c
*Pep-D*	b	b	b	b^98^,a
*Pgk*	a^98^,b	a^97^,b	a	a
*Pgm*	c^98^,b	c^90^,e^9^,b	c^97^,a	c
Sordh*	c	d^45^,a^23^,b^20^,c	b	d
*Tpi*	b	b^98^,a^1^,c	b^83^,c	b

For polymorphic loci, the frequencies of all but the rarer/rarest alleles are expressed as percentages and shown as superscripts (allowing the frequency of each rare allele to be calculated by subtraction from 100%). The maximum sample size is shown in brackets for each taxon. * Loci diagnostic for distinct clades. The following loci were invariant: *Argk*, *Enol*, *Fdp*, *Gp1*, *Gp2*, *Ipp*, and *Pk*.

### Quantitative Allozyme Perspectives on Population Structure

Statistical tests for each of the 19 sites found no evidence for genotypic departure from Hardy Weinberg expectations and for linkage disequilibrium. We therefore considered it appropriate to undertake site-based analyses of population genetic structure for those taxa which have been sampled from multiple spring complexes such as clades S and C (the two clade N sites represent very distinctive genetic lineages; Fig. 4).

Both statistical analyses of between-site heterogeneity (Table S3, Table S4) recovered (Geneland) or supported (GENEPOP) the same number of subpopulations in each taxon, namely k = 3 for the eight sites representing clade S and k = 8 for the eight included sites in clade C (Emerald site excluded due to its low sample size). Moreover, not only were more subpopulations identified in clade C, the levels of genetic differentiation among these subpopulations were almost always considerably higher than in clade S (Fig. 4; see also F_ST_ values in Table S3 and Table S4).

## Discussion

The GAB mound springs in Australia’s arid zone are proving to be complex and dynamic ecological systems with multiple layers to their history. The endemic fauna that are restricted to these springs are considered to be ecologically and evolutionarily relictual from a time when central Australia comprised a wet and temperate habitat [15], [28]. Our observations of deep phylogenetic divergences within the mound springs endemic isopod, *P. latipes*, challenge the current status of *Phreatomerus* as a monotypic genus with a wide distribution throughout the Lake Eyre mound springs. Striking phylogeographic structure at a regional scale has shown that multiple levels of genetic diversity exist in this taxon throughout the region, at ancient through to more recent time-scales. In light of the complexity encountered, we examined three aspects of the evolutionary history of *Phreatomerus* with respect to the geological and climatic history of the Lake Eyre catchment. These were: a) the deep, older, relationships amongst three primary phylogenetic clades (C, S, N), where the distribution of historical lineages was examined, b) the intermediate genetic relationships, where nine sub-clades (I-IX) were examined with respect to the influence of a changing landscape on lineage divergence, and c) the potential influence of Lake Eyre drainages on gene flow between *P. latipes* sub-clades.

### Phylogenetic Relationships Among Major Clades

The oldest relationships amongst the mound spring faunas pose a challenge for reconstructing historic biogeography and understanding species distributions because of the complex climatic and geological history of the region. In particular, repeated climatic changes in aridification since the mid-late Miocene are likely to have regularly overwritten some previous features of the landscape, especially for aquatic habitats. Here we identified three major clades, concordant for both mtDNA (mean estimate 18.7 (12.2–25.8 95% HPD)–13.5 (8.0–19.6 95% HPD) million years divergent) and allozyme profiles (4 diagnostic allozyme differences between each). These clades (C, S, N) were found to be completely allopatric. Nevertheless, clade C included an intriguing geographic outlier, namely the Freeling spring complex. As the most northern spring complex, Freeling is geographically much closer to the spring complexes within the Umbum Creek drainage, albeit in the separate Neales Creek drainage. This pattern, observed in both mtDNA and allozymes, is inconsistent with a simple hypothesis of multiple species originating from a single common ancestor and subsequent isolation by distance. Instead it suggests a more complex phylogeographic history for clade C and, by inference, clade N.

The geological history of the mound springs dates back to the late Pliocene, early Pleistocene [9], [64] with the oldest ‘known’ springs estimated to be 740±120 thousand years old (Elizabeth Springs [64]). Activation and formation of the springs is considered to have occurred up to 1–2 million years ago (mya) at most [65], [66], making the GAB mound springs relatively young in the history of the region. Current date estimates among the three major clades placed their divergence firmly into the Miocene era, with the earliest divergence between clade C and clades N+S given a mean estimate of 18.7 (12.2–25.8 95% HPD) mya and subsequent splits between N and S given a mean estimate of 15.7 (10.2–21.5 95% HPD) mya. Both sets of dates precede formation of springs and the stony deserts by more than 15 mya, suggesting that the origins of these three lineages pre-date major aridification events in the Lake Eyre region. Instead, it is likely that these major clades represent an ancient and diverse fauna in Australia’s arid zone prehistory. Subsequent regional isolation of fauna is thought to have led to the relict populations we now see (see further below). Evidence for such an hypothesis has been presented for other endemic mound springs taxa (i.e. amphipods [15] and snails [28]), including, ancient phylogenetic connections with other relictual aquatic regions of Australia [15] that fit with our date estimates. Those authors have proposed that during the Miocene, central Australia comprised a warm-wet habitat that extended throughout what is currently known as the arid zone and comprised a rich fauna, both aquatic and terrestrial. Subsequent major aridification events [1] are thought to have led to widespread extinction events except in those instances where pockets of remnant habitat retained a relict fauna [12], [13], [14], [15]. We consider that this hypothesis also applies to the endemic phreatoicid found in the mound springs in which three lineages remain extant today, but where many now-extinct lineages probably once existed. A scenario of widespread genetic lineages formerly found throughout the Lake Eyre region also explains the occurrence of clade C individuals in both the geographically northernmost spring complex (Freeling) and in central Lake Eyre springs.

### Origins of mtDNA Sub-clades

Recent work on spring snails in Lake Eyre mound springs has led to a proposal known as the ‘stranded in desert springs’ hypothesis which states that widespread spring endemics have historically become stranded in GAB spring refugia due to Miocene aridification [28]. Here we have been able to examine this hypothesis in further detail for another spring endemic invertebrate, *P. latipes* with contrasting results. In addition to three major relict clades, indicated by both sets of markers, we also observed nine well-supported mtDNA lineages (i.e. clade C: I Strangways/Francis, II Freeling, III Strangways-only, IV Central (Coward + Billa Kalina + Beresford + Lake Eyre South), clade S: VIII Wangianna and IX Hermit Hills, clade N: V, VI and VII Neales River). Each of the mtDNA sub-clades within the three major clades displayed similarity in coalescent dates (8.8 (5.7–12.6 95% HPD)–2.2 (0.2–5.1 95% HPD) mya, excluding Neales sub-clade VI which had a date of 12.3 (7.2–17.7 95% HPD) mya). We also observed a match between geographic locality and genetic structure within the Lake Eyre mound spring system, with each of these clades corresponding largely to the grouping of spring complex. This historical grouping defines spatially close springs (1 km between individual springs) situated within a similar geomorphic setting and relative position to Lake Eyre. However, two exceptions were observed; clade N (also the Neales complex), which was split into three sub-clades, suggesting substantial sub-structuring within the Neales complex (concordant with the allozyme data), plus two sympatric sub-clades at Strangways (not detectable allozymically). Despite several modest differences between the allozyme and mtDNA perspectives for some spring complexes, a general concordance was observed between all of the sub-clades and the natural groupings of spring complexes for both allozyme data and mtDNA. We therefore consider our findings to be robust enough to support the hypothesis that the intervening landscape and isolation between spring complexes has played an important role in the evolutionary patterns observed where strong genetic structure exists. In particular, these sub-clades likely reflect the geomorphic proximity and similarity in physico-chemical composition of the area, but overall a climate and landscape mediated contraction and subsequent diversification event during the history of *P. latipes* in the mound springs is predicted to reflect its present day divergences.

A major period of aridification 15–7 mya and a ‘return to wet’ is thought to have occurred around 6 mya in the Australian arid zone [1]. This period is invoked widely as a key timescale during which habitats and relictual populations were isolated and subsequently trapped *in situ*, notably the calcrete aquifers in Western Australia [14], [32], riparian woodland [1] and mesic forest habitats [13]. Here we see that the 12.3 (7.2–17.7 95% HPD) and 8.8 (5.7–12.6 95% HPD)–2.2 (0.2–5.1 95% HPD) mya coalescent timescale for the diversification of the nine sub-clades of *P. latipes* coincides with dates listed for aridification, a return to wet and subsequent Pleistocene aridification. However, the sub-clade dates are substantially earlier than those estimated for a) formation of mound springs (∼1 mya) and the stony deserts (4-2 mya) [9], [64] and also the b) intraspecific divergences of spring snails (0.8–1.5 mya) [28]. We take into account that the latter could also be due to a difference in the calibration times where a Protostomia *COI* substitution rate of 1.76% per million years was used to calibrate the clock tree [67] for snails compared with the standard arthropod mtDNA molecular clock of 2.3% divergence per million years [58] used here. However, the time differences are substantial and most likely reflect some real differences in divergence timing. Therefore, we hypothesise that *P. latipes* was stranded in wetland habitats in the Lake Eyre region earlier than that estimated for snails and that events spanning the mid Miocene-Pleistocene were probably most influential. Under this scenario each sub-clade in *P. latipes* is thought to represent a contraction of populations within the nine sub-clades to regional habitats (i.e. rivers, wetlands, etc.) during mid-Miocene aridification, at which time a contraction of aquatic habitats and a fall in water tables are likely to have occurred [65]. However, the true climatic conditions around 14 mya are unknown and this time period is often referred to as the “Hill Gap” due to the lack of information in the geological and sedimentary record [1], [68], [69]. A subsequent return to wet during the Pliocene probably led to substantial lineage expansion. We consider direct colonisation of the mound springs to be a consequence of the final contraction of, and adaptation to the newly isolated habitat for, these freshwater limited *P. latipes* as the arid zone habitat became more inhospitable, particularly during the 100 ka glacial cycles of the late Pleistocene. Subsequently, these fragmented pockets of relictual aquatic habitat were able to maintain large populations of invertebrates and thus have more recently promoted and maintained genetic differentiation across the arid landscape. While our date estimates are consistent with the beginning of lineage diversification following Miocene aridification of Australia, it must be noted that our molecular clock analyses were limited by the use of a standard rate calibration [58], due to the absence of fossils that could be used to calibrate a molecular clock. The only fossil phreatoicid that currently exists is the distant relative *Protamphisopus wianamattensis* [48]. Nevertheless, a substantially reduced (2−>8 fold) slower rate than our standard would be required to obtain lineage diversification consistent with the proposed ∼1 my time period for the formation of mound springs.

### Importance of the Surface Drainages: Aquatic Connections between Sub-clades

For desert spring species that lack terrestrial dispersal abilities and are unable to disperse passively via phoresy [70], [71], river drainages may provide vital dispersal highways between these otherwise fragmented environments [38]. A major influence on the population structure of springs and their fauna is likely to be river catchments that flow intermittently inland towards Lake Eyre. A number of major river drainages flow among southern Lake Eyre mound springs and many contain catchment-specific mtDNA sub-clades, e.g. Margaret and Warriner Creeks (I and III Strangways/Francis, IV Coward (including Billa Kalina, Beresford, Lake Eyre South), Neales Creek (II Freeling); a number of rivers around the Hermit Hills drainage (i.e. VIII Wangianna, IX Hermit Hills) and Umbum Creek (V, VI, and VII Neales River). Each of these rivers could possibly link and structure springs as seen in other mound springs invertebrates [23], [27] and predicted by Murphy *et al*. [26] for *P. latipes*. With few exceptions, we observed little evidence in either dataset of present day gene flow (i.e. shared haplotypes) especially between major clades, which were completely allopatric, or between sub-clades. The only evidence of contemporary migration across the desert we observed was between the Central spring complexes: Francis Swamp ↔ Strangways complexes (sub-clade I, haplotype FS1) and Beresford ↔ Coward complexes. River mediated gene flow is a likely explanation for these shared haplotypes with individuals able to move between spring complexes during periods of major flooding. Interestingly, population genetic structure remains very strong amongst these populations based on allozymes and mostly allopatric mtDNA sub-clades suggesting that typically such migration events are likely to be rare and either do not contribute substantially to the population’s gene pool or mostly involve males.

### Conservation Implications

Here we have identified three major layers of evolutionary diversity in *P. latipes*, one operating at a broad regional scale (i.e. three clades C, S, and N) and the others at the mid scale with nine sub-clades (i.e. I-IX) and at the local population level of rivers. While future work will investigate the latter in more detail, all of these levels are relevant for conservation of biodiversity within the nationally protected mound spring community around Lake Eyre. Our results are particularly pertinent given the general phylogeographic patterns observed here for *P. latipes* mirror those displayed in co-occurring mound spring endemics such as amphipods [15], [28] and wolf spiders [27]. For example, amongst amphipods [15] three major geographic clades were also observed: southern/central (Clade A), northern (Clade B) and Strangways and Francis Swamp (Clade C), each of which contained sub-clades of likely candidate species (n = 4, n = 3 and n = 1, respectively) (after Murphy *et al*. [15]). Both of these taxon groups have revealed little to no evidence of gene flow at large geographic scales, suggesting that most mound spring endemics disperse between populations, sometimes at moderate distances (e.g. Francis-Strangways, Beresford-Coward complexes), probably along major river drainages [15], [23], [24], [27], [28]. The presence of many genetically distinct lineages, and in some cases sympatric lineages within a single spring, strongly suggest extraordinary diversity at multiple geographic scales. Overall therefore, a case is emerging for phylogeographic management units throughout the Lake Eyre supergroup that aim to conserve both taxa and springs that have a shared phylogeographic history, which has been suggested for North American spring systems [16]. Extinction and reactivation of springs is a naturally occurring phenomenon, but water extraction from the GAB for mining and pastoral activities has reduced the flow of many Lake Eyre springs in large areas. For conservation purposes, identification of broad geographic regions would prevent micro-management of individual springs and would instead encourage large-scale preservation of bioregions with some flexibility for stochastic habitat loss amongst the individual mound spring groups.

Taken together, our mtDNA and allozyme data argue strongly that clades C, S and N should each be regarded as separate species. The number of independent and concordant taxonomic characters (four allozyme loci plus mtDNA), levels of genetic divergence encountered (mtDNA divergence estimates >15 mya), and overall geographic patterns encountered are, when combined, consistent with the standard operating criteria employed under most modern species concepts (including biological, evolutionary, and phylogenetic versions; [72]). Morphological examination has revealed some evidence for morphological differences concordant with the three major clades, but these data are still preliminary (R. King pers. comm.). Importantly, our preliminary allozyme data for clade N revealed levels of allozyme divergence between the only two springs for which frozen tissues were available (i.e. Outside and Fountain) that were comparable to those found between the three candidate species. Given these two spring populations also fell into different mtDNA sub-clades, our genetic data also infer that additional candidate species may be present in the little-sampled northern mound springs surrounding Lake Eyre. Further intensive sampling, followed by additional mtDNA and nuclear genetic characterization, is required to test this and other working hypotheses, including the phylogeographic history that led to the genetic similarity of the northern Freeling and central Lake Eyre spring complexes.

Regardless of how many species will ultimately be described, all will qualify as short-range endemics [73] and be restricted solely to certain spring complexes of mound springs. Further, most relatively widespread species are likely to comprise two or more distinctive genetic lineages and significant geographic sub-structuring. These findings already have an immediate impact on the conservation perspective afforded to *P. latipes*, which only comes in the form of protection at the spring community level, given the genus can no longer be regarded as monotypic and the “species” no longer considered widespread. Clearly, the continuation of the mound springs as protected areas of high conservation value is essential for the continued existence of its unique diversity of freshwater endemics.

### Conclusions

Overall, we conclude that the phylogenetic history of the ancient relict taxon, *P. latipes*, has highlighted the process of aridification in central Australia from a time when it previously comprised a wet and swamp-like environment. The genus reflects a diverse fauna that existed during the early Miocene and appears to have been regionally restricted. Subsequent aridification events have led to substantial contraction of the habitat, and isolation of *P. latipes* at a local geographic scale has remained stable, with only some evidence of long-distance dispersal by extant populations indicating that they represent relictual species. The multiple layers of phylogeographic history that are exemplified by *P. latipes* have been similarly observed in other mound springs taxa. This concordance suggests that major climate events and landscape structure have clearly shaped the high levels of diversity and endemism seen here, in particular, isolation of springs. Conservation of the GAB mound springs habitats is of utmost importance for preserving a fauna that reflects the ancient history of the Australian arid landscape.

## Supporting Information

Figure S1 Consensus phylogeny estimated using a Bayesian approach that employed the GTR +I +G model of nucleotide evolution for haplotypes of *Phreatomerus latipes*. The tree is rooted with three outgroups. Posterior probability support is listed as a percentage next to the corresponding node.
(PDF)

Table S1 Data table containing locality data for *Phreatomerus latipes* including the collection number (GAB#), the number of specimens sequenced from a particular location (n), the number of haplotypes (*h*) and their names (name and frequency in parentheses) and their respective position in the final phylogenetic analysis from Figure 1 (clades = S, Southern, C, Central, Northern, and sub-clades = I–IX).
(DOCX)

Table S2 Estimates of time since most recent common ancestor (time per million years) of haplotypes from individual sub-clade/clades based on five coalescent models using a Bayesian coalescent approach with BEAST [57].
(DOCX)

Table S3 Summary of between-site assessments of heterogeneity for clade S sites. The lower triangle indicates which pairwise comparisons among sites were statistically significant across all loci, calculated using Fisher’s method; * = 0.01<P<0.05; *** = P<0.001; ns = not significant (exact P values, after Bonferroni correction, as calculated using GENEPOP). The upper triangle presents the FST value obtained using Geneland. None of the analyses found any statistical evidence of heterogeneity among the five sites included in “all other sites”.
(DOCX)

Table S4 Summary of between-site assessments of heterogeneity for clade C sites (excluding Emerald).
(DOCX)
