# Supplementary figures and images for: Desert Springs: Deep Phylogeographic Structure in an Ancient Endemic Crustacean (Phreatomerus latipes)
Source: PLoS One. 2012 Jul 17;7(7):e37642. doi: 10.1371/journal.pone.0037642 (PMC3398905; doi:10.1371/journal.pone.0037642)

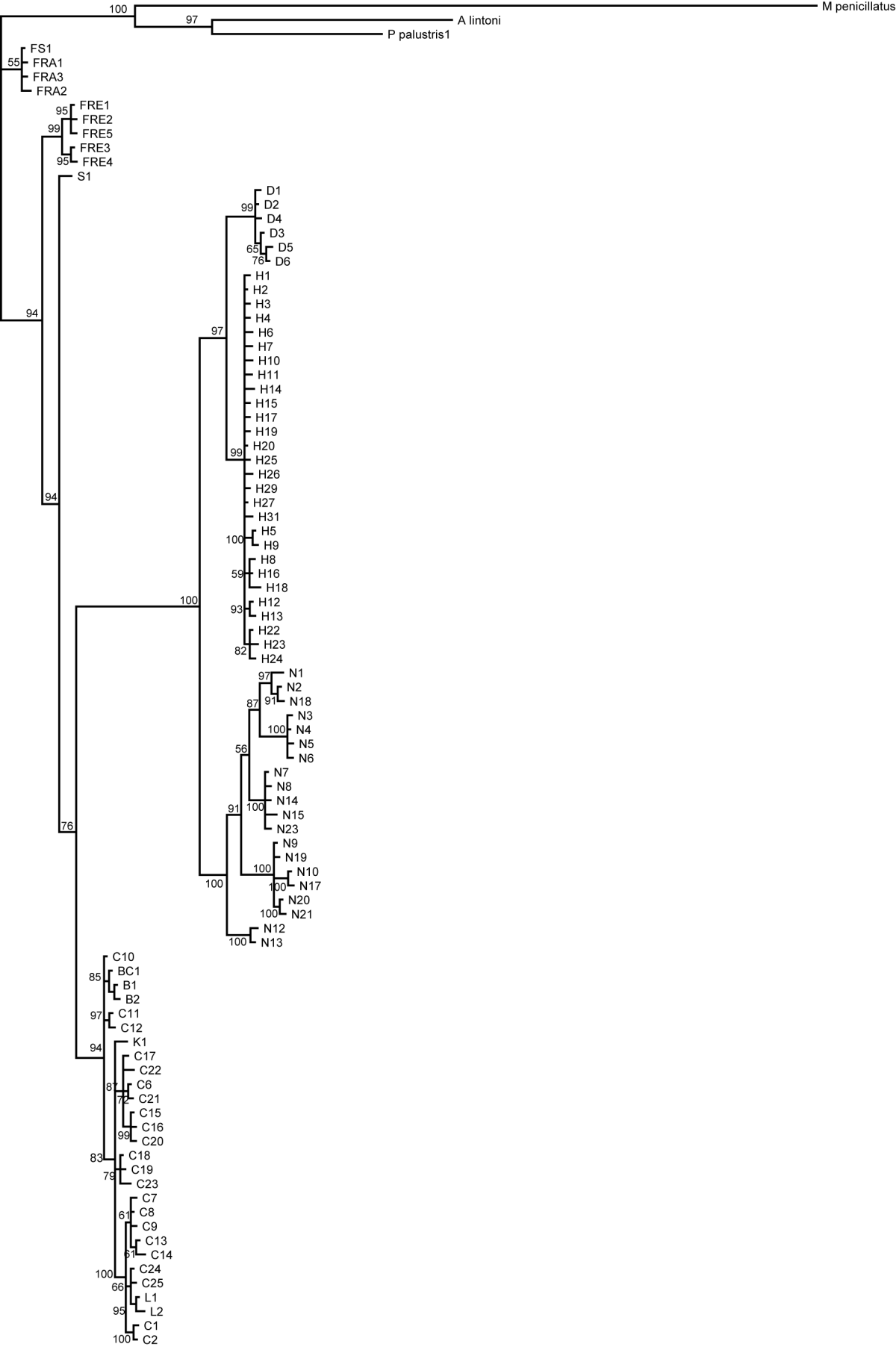

Supplement: Figure S1 — Consensus phylogeny estimated using a Bayesian approach that employed the GTR +I +G model of nucleotide evolution for haplotypes of Phreatomerus latipes. The tree is rooted with three outgroups. Posterior probability support is listed as a percentage next to the corresponding node. (PDF) [file pone.0037642.s001.pdf]
