# Supplementary material for: Desert Springs: Deep Phylogeographic Structure in an Ancient Endemic Crustacean (Phreatomerus latipes)
Source: PLoS One. 2012 Jul 17;7(7):e37642. doi: 10.1371/journal.pone.0037642 (PMC3398905; doi:10.1371/journal.pone.0037642)
Supplement: Table S1 — Data table containing locality data for Phreatomerus latipes including the collection number (GAB#), the number of specimens sequenced from a particular location (n), the number of haplotypes (h) and their names (name and frequency in parentheses) and their respective position in the final phylogenetic analysis from Figure 1 (clades = S, Southern, C, Central, Northern, and sub-clades = I–IX). (DOCX) [file pone.0037642.s002.docx]

| **River drainage** | **Spring Complex** | **Spring Group** | **Spring** | **Latitude** | **Longitude** | **GAB #** | **n** | ***h*** | **Haplotype** | **Sub-clade** |
| --- | --- | --- | --- | --- | --- | --- | --- | --- | --- | --- |
|  |  |  |  |  |  |  |  |  |  |  |
| ***Warriner Creek*** | ***Beresford*** | Beresford | BBH | -29.26407 | 136.66373 | 348 | 5 | 1 | B1 (5) | C-IV |
|  |  | Warburton | BWSC | -29.27322 | 136.6713 | 215 | 6 | 3 | B1 (4), 2, BC1 |  |
|  |  |  |  |  |  | **TOTAL** | **11** |  |  |  |
| ***Margaret Creek*** | ***Coward*** | Blanche | BC5 | -29.4509 | 136.86285 | 672 | 8 | 3 | C8 (6), 13, 14 | C-IV |
|  |  |  | BCP | -29.45292 | 136.85872 | 356 | 10 | 2 | C8 (9), 14 |  |
|  |  | Buttercup | CBS | -29.48116 | 136.89476 | 171 | 10 | 2 | C1 (9), 2 |  |
|  |  | Coward | CCS2 | -29.40061 | 136.7942 | 389 | 8 | 4 | BC1 (4), 10 (2), 11, 12 |  |
|  |  | Horse East | CHS1 | -29.4889 | 136.92151 | 762 | 14 | 2 | C24 (11), 25 (3) |  |
|  |  | Horse West | CHW | -29.48652 | 136.91293 | 371 | 8 | 3 | C7, 8 (6), 9 |  |
| ***Warriner Creek*** |  | Elizabeth North | CENC | -29.35725 | 136.76863 | 687 | 10 | 2 | C15 (8), 16 (2) |  |
|  |  |  | CNE94 | -29.35311 | 136.76758 | 759 | 8 | 1 | C17 (8) |  |
|  |  | Elizabeth South | CESA | -29.36104 | 136.77722 | 699 | 8 | 2 | C6 (5), 21 (3) |  |
|  |  |  | CESB | -29.36175 | 136.77756 | 700 | 9 | 1 | C6 (9) |  |
|  |  |  | CESE | -29.35909 | 136.77579 | 703 | 3 | 2 | C6 (2), 20 |  |
|  |  | Jersey | CJSA | -29.34129 | 136.75642 | 722 | 5 | 1 | C22 (5) |  |
|  |  |  | CJSD | -29.34179 | 136.757714 | 724 | 2 | 1 | C22 (2) |  |
|  |  | Kewson Hill | CKHA | -29.36882 | 136.78319 | 748 | 14 | 1 | C23 (14) |  |
|  |  |  | CKHK | -29.3671 | 136.7841 | 757 | 9 | 2 | C18 (8), 19 |  |
|  |  |  |  |  |  | **TOTAL** | **126** |  |  |  |
|  | ***Strangways*** | Strangways | STS |  |  | I18* | 7 | 1 | S1 (7) | C-III |
|  |  |  | STS1 | -29.15969 | 136.54875 | 339 | 16 | 1 | FS1 (16) |  |
|  |  |  | STSC | -29.15972 | 136.55116 | 331 | 11 | 2 | S1 (11) |  |
|  |  |  | STSD | -29.16078 | 136.55132 | 289 | 4 | 1 | S1 (4) |  |
|  |  |  |  |  |  | **TOTAL** | **38** |  |  |  |
|  | ***Billa Kalina*** |  | KBK | -29.487 | 136.449 | I16* | 6 | 1 | K1 (6) | C-IV |
|  |  |  |  |  |  | **TOTAL** | **6** |  |  |  |
|  | ***Lake Eyre South*** | Emerald | LES | -29.38322 | 137.06432 | 770 | 8 | 2 | L1 (5), 2 (3) | C-IV |
|  |  |  |  |  |  | **TOTAL** | **8** |  |  |  |
| ***Neales Creek*** | ***Freeling*** | Freeling | EFS |  |  | I2*  I22* | 8 | 3 | FRE1 (6), 2, 3 | C-II |
|  |  |  | EFS1 | -28.071 | 135.905 | 1594 | 5 | 2 | FRE1 (4), 5 |  |
|  |  |  | EFS21 |  |  | 1600 | 2 | 2 | FRE1, 4 |  |
|  |  |  | EFS29 |  |  | 1629 | 4 | 1 | FRE1 (4) |  |
|  |  |  |  |  |  | **TOTAL** | **19** |  |  |  |
| ***Warriner Creek*** | ***Francis Swamp*** | Francis Swamp | FFS |  |  | I19* | 1 | 1 | FS1 | C-I |
|  |  |  | FFSC | -29.17235 | 136.29379 | 258  260 | 11 | 1 | FS1 (11) |  |
|  |  |  | FFSF |  |  | 194 | 2 | 2 | FRA2, 3 |  |
|  |  |  | FFSG | -29.17525 | 136.2961 | 320  321 | 9 | 1 | FRA1 (9) |  |
|  |  |  |  |  |  | **TOTAL** | **23** |  |  |  |
| ***Hermit Hills catchment*** | ***Hermit Hills*** | Bopeechee | HBO |  |  | I10* | 1 | 1 | H8 | S-VIII |
|  |  |  | HBO004 | -29.606 | 137.387 | 547 | 4 | 4 | H2, 16, 17, 18 |  |
|  |  | Dead Boy | HDB |  |  | I9* | 1 | 1 | H27 |  |
|  |  | Finnis | HF | -29.582 | 137.471 | I3* | 1 | 1 | H12 |  |
|  |  | Hermit Hills | HH |  |  | I7* | 2 | 2 | H11, 14 |  |
|  |  |  | HHS039 |  |  | 661 | 5 | 2 | H20 (4), 26 |  |
|  |  |  | HHS188 | -29.574 | 137.430 | 583 | 4 | 3 | H2, 19, 20 (2) |  |
|  |  | Old Finnis | HOF058 | -29.587 | 137.447 | 542 | 4 | 4 | H2, 10, 14, 15 |  |
|  |  |  | HOF063 |  |  | 487 | 5 | 2 | H2 (4), 11 |  |
|  |  |  | HOF081 |  |  | 533 | 4 | 2 | H2 (3), 14 |  |
|  |  |  | HOFSDA |  |  | 792 | 5 | 2 | H2 (3), 10 (2) |  |
|  |  | Old Woman | HOW009 | -29.597 | 137.451 | 472 | 5 | 2 | H2, 10 (4) |  |
|  |  |  | HOW012 |  |  | 483 | 3 | 1 | H10 (3) |  |
|  |  |  | HOW026 |  |  | 510 | 4 | 3 | H10, 12, 13 (2) |  |
|  |  | Sulfuric | HSS011 | -29.608 | 137.402 | 594 | 3 | 1 | H20 (3) |  |
|  |  |  | HSS012 |  |  | 777 | 4 | 2 | H2 (3), 29 |  |
|  |  |  | HSS016 |  |  | 607 | 4 | 4 | H22, 23, 24, 25 |  |
|  |  |  | HSS048 |  |  | 638 | 4 | 3 | H15, 20 (2), 26 |  |
|  |  | Venables | HVS | -29.678 | 137.361 | I11* | 1 | 1 | H31 |  |
|  |  | West Finnis | HWF002a | -29.595 | 137.409 | 452 | 5 | 5 | H2, 6, 7, 8, 9 |  |
|  |  |  | HWF043 |  |  | 436 | 3 | 2 | H1 (2), 3 |  |
|  |  |  | HWF045 |  |  | 432 | 5 | 2 | H1 (2), 2 (3) |  |
|  |  |  | HWF048 |  |  | 446 | 5 | 3 | H2 (2), 4, 5 (2) |  |
|  |  |  |  |  |  | **TOTAL** | **82** |  |  |  |
| ***Umbum Creek*** | ***Neales River*** | Brinkley | NBS | -28.502 | 136.306 | 958  1697 | 20 | 4 | N3 (4), 4 (12), 5 (3), 6 | N-VI |
|  |  | Fanny | NFS2 | -28.32349 | 136.24037 | 1663 | 2 | 2 | N14, 15 |  |
|  |  |  | NFS3 | -28.32349 | 136.24037 | 967 | 8 | 2 | N14 (3), 15 (5) |  |
|  |  | Fountain | NTF | -28.348 | 136.28271 | 952  I20* | 19 | 3 | N1 (5), 2 (13), 18 |  |
|  |  | Hawker | NHS1 | -28.442876 | 136.19092 | 944 | 14 | 2 | N7 (13), 8 |  |
|  |  |  | NHS4 | -28.42209 | 136.18782 | 999 | 12 | 2 | N7 (11), 23 |  |
|  |  | Milne | NMI | -28.26484 | 136.07664 | 977  1913 | 22 | 2 | N12 (5), 13 (17) |  |
|  |  | Outside | NOS |  |  | I21 | 2 | 1 | N12 (2) | N-V |
|  |  |  | NOS1 | -28.26256 | 136.19849 | 995 | 1 | 1 | N12 |  |
|  |  |  | NOS3 | -28.26587 | 136.20253 | 1014  1662 | 8 | 1 | N12 (8) |  |
|  |  | Twelve Mile | NTM | -28.30586 | 136.25781 | 988 | 23 | 6 | N9 (6), 10 (13), 17, 19, 20, 21 | N-VII |
|  |  |  |  |  |  | **TOTAL** | **131** |  |  |  |
|  | ***Wangianna*** | Davenport | WDS | -29.66176 | 137.58691 | 15* | 3 | 2 | D2 (2), 4 | S-IX |
|  |  |  | WDSC | -29.66399 | 137.58899 | 807 | 8 | 2 | D1, 2 (7) |  |
|  |  |  | WDSE | -29.66233 | 137.58522 | 809 | 15 | 3 | D1 (5), 2 (9), 3 |  |
|  |  | Welcome | WWS | -29.674 | 137.825 | I4* | 6 | 3 | D2 (4), 5, 6 |  |
|  |  |  |  |  |  | **TOTAL** | **32** |  |  |  |

* Specimens sampled in 1985
