# Supplementary material for: Desert Springs: Deep Phylogeographic Structure in an Ancient Endemic Crustacean (Phreatomerus latipes)
Source: PLoS One. 2012 Jul 17;7(7):e37642. doi: 10.1371/journal.pone.0037642 (PMC3398905; doi:10.1371/journal.pone.0037642)
Supplement: Table S2 — Estimates of time since most recent common ancestor (time per million years) of haplotypes from individual sub-clade/clades based on five coalescent models using a Bayesian coalescent approach with BEAST [57]. (DOCX) [file pone.0037642.s003.docx]

| Sub-clade/clade | Constant | | | Exponential | | | Logistic | | | Expansion | | | Yule | | |
| --- | --- | --- | --- | --- | --- | --- | --- | --- | --- | --- | --- | --- | --- | --- | --- |
|  | mean | 95% HPD | | mean | 95% HPD | | mean | 95% HPD | | mean | 95% HPD | | mean | 95% HPD | |
|  |  | lower | upper |  | lower | upper |  | lower | upper |  | lower | upper |  | lower | upper |
| I Francis/Strangways | 2.9 | 0.7 | 5.5 | 2.8 | 0.7 | 5.4 | 3.0 | 1.0 | 5.6 | 3.0 | 0.9 | 5.3 | 3.2 | 0.9 | 6.0 |
| II Freeling | 4.7 | 1.5 | 8.4 | 4.8 | 1.6 | 8.5 | 4.8 | 2.1 | 8.1 | 4.7 | 2.0 | 8.1 | 5.2 | 1.9 | 9.1 |
| IV Coward | 7.9 | 5.0 | 11.4 | 8.0 | 5.2 | 11.5 | 8.0 | 5.1 | 11.1 | 7.6 | 5.0 | 10.6 | 8.8 | 5.7 | 12.6 |
| clade C (I+II+III+IV) Total | 27.4 | 18.8 | 38.3 | 25.8 | 16.9 | 34.5 | 20.1 | 12.4 | 29.8 | 25.0 | 16.1 | 34.1 | 18.7 | 12.2 | 25.8 |
| V [= V Neales1] | 1.6 | 0.1 | 3.9 | 1.7 | 0.1 | 4.1 | 2.0 | 0.2 | 4.4 | 1.9 | 0.3 | 4.0 | 2.2 | 0.2 | 5.1 |
| VI [= V Neales2] | 14.4 | 8.2 | 20.9 | 14.1 | 8.6 | 20.4 | 11.4 | 6.5 | 16.9 | 12.8 | 7.6 | 18.7 | 12.3 | 7.2 | 17.7 |
| VII [= V Neales3] | 3.9 | 1.6 | 6.6 | 4.0 | 1.7 | 6.7 | 4.2 | 2.0 | 6.7 | 3.9 | 1.9 | 6.1 | 4.5 | 2.1 | 7.3 |
| clade N (V+VI+VII) Total | 19.5 | 13.1 | 27.0 | 18.9 | 13.0 | 25.7 | 15.1 | 9.4 | 21.7 | 17.5 | 11.9 | 24.2 | 15.7 | 10.2 | 21.5 |
| VIII Davenport | 2.6 | 0.7 | 4.9 | 2.7 | 1.0 | 5.1 | 3.4 | 1.2 | 5.8 | 3.0 | 1.3 | 5.1 | 3.8 | 1.2 | 6.7 |
| IX Hermit Hills | 5.5 | 3.2 | 8.2 | 5.8 | 3.3 | 8.7 | 6.1 | 3.7 | 8.5 | 5.3 | 3.4 | 7.7 | 6.9 | 4.1 | 10.0 |
| clade S (VIII + IX) Total | 15.7 | 9.0 | 23.7 | 15.3 | 8.9 | 22.1 | 12.7 | 7.6 | 19.3 | 14.6 | 8.4 | 21.5 | 13.5 | 8.0 | 19.6 |
