# Supplementary material for: Desert Springs: Deep Phylogeographic Structure in an Ancient Endemic Crustacean (Phreatomerus latipes)
Source: PLoS One. 2012 Jul 17;7(7):e37642. doi: 10.1371/journal.pone.0037642 (PMC3398905; doi:10.1371/journal.pone.0037642)
Supplement: Table S3 — Summary of between-site assessments of heterogeneity for clade S sites. The lower triangle indicates which pairwise comparisons among sites were statistically significant across all loci, calculated using Fisher’s method; * = 0.01<P<0.05; *** = P<0.001; ns = not significant (exact P values, after Bonferroni correction, as calculated using GENEPOP). The upper triangle presents the FST value obtained using Geneland. None of the analyses found any statistical evidence of heterogeneity among the five sites included in “all other sites”. (DOCX) [file pone.0037642.s004.docx]

| **Spring**  **complex** | **Subpopulation** | **Welcome** | **Davenport** | **Venables** | **All other sites** |
| --- | --- | --- | --- | --- | --- |
| **Davenport** | **Welcome** | - | 0.605 | 0.690 | 0.605 |
|  | **Davenport** | *** | - | 0.029 | 0.036 |
| **Venables** | **Venables** | *** | ns | - | 0.053 |
|  | **All other sites** | *** | * | * | - |
