# Supplementary material for: Desert Springs: Deep Phylogeographic Structure in an Ancient Endemic Crustacean (Phreatomerus latipes)
Source: PLoS One. 2012 Jul 17;7(7):e37642. doi: 10.1371/journal.pone.0037642 (PMC3398905; doi:10.1371/journal.pone.0037642)
Supplement: Table S4 — Summary of between-site assessments of heterogeneity for clade C sites (excluding Emerald). (DOCX) [file pone.0037642.s005.docx]

| **Spring complex** | **Subpopulation** | **Coward** | **Blanche Cup** | **Elizabeth** | **Beresford** | **Billa Kalina** | **Strangways** | **Francis** | **Freeling** |
| --- | --- | --- | --- | --- | --- | --- | --- | --- | --- |
| **Coward** | **Coward** | - | 0.210 | 0.469 | 0.833 | 0.738 | 0.749 | 0.784 | 0.788 |
|  | **Blanche Cup** | *** | - | 0.373 | 0.664 | 0.491 | 0.552 | 0.596 | 0.645 |
|  | **Elizabeth** | *** | *** | - | 0.791 | 0.726 | 0.702 | 0.750 | 0.768 |
| **Beresford** | **Beresford** | *** | *** | *** | - | 0.923 | 0.847 | 0.875 | 0.906 |
| **Billa Kalina** | **Billa Kalina** | *** | *** | *** | *** | - | 0.569 | 0.683 | 0.824 |
| **Strangways** | **Strangways** | *** | *** | *** | *** | *** | - | 0.198 | 0.728 |
| **Francis** | **Francis** | *** | *** | *** | *** | *** | *** | - | 0.787 |
| **Freeling** | **Freeling** | *** | *** | *** | *** | *** | *** | *** | - |
